# ?*Amphictis* (Carnivora, Ailuridae) from the Belgrade Formation of North Carolina, USA

**DOI:** 10.7717/peerj.9284

**Published:** 2020-07-08

**Authors:** Jon Baskin, Edwin Dickinson, John DuBois, Henry Galiano, Adam Hartstone-Rose

**Affiliations:** 1 Department of Biological and Health Sciences, Texas A&M University—Kingsville, Kingsville, TX, United States of America; 2 UT Jackson School of Geoscience, University of Texas at Austin, Austin, TX, United States of America; 3 Department of Biological Sciences, North Carolina State University, Raleigh, NC, United States of America; 4 Benson, NC, United States of America; 5 Maxilla & Mandible, Ltd., New York, NY, United States of America

**Keywords:** ?*Amphictis*, Carnivora, Ailuridae, Belgrade formation, North Carolina

## Abstract

Miocene terrestrial mammals are poorly known from the Atlantic Coastal Plain. Fossils of the Order Carnivora from this time and region are especially rare. We describe a carnivoran mandible with a p4 from the late Oligocene or early early Miocene Belgrade Formation in Jones County, North Carolina. Comparisons are made with carnivoran jaws with similar premolar and molar lengths from the late Oligocene and Miocene of North America and Eurasia. These indicate that the North Carolina jaw is assignable to the Ailuridae, a family whose only living member is the red panda. The jaw is tentatively referred to *Amphictis*, a genus known elsewhere from the late Oligocene and early Miocene of Europe and the early Miocene (Hemingfordian) of North America.

The North Carolina mandible compares best with the late Oligocene (MP 28) *Amphictis ambiguus* from Pech du Fraysse, France, the oldest known member of the Family Ailuridae, and with the early Miocene (MN 1–MN 2a) *A. schlosseri* from southwestern Germany. This identification is compatible with a late late Arikareean (Ar4, early Miocene, MN 2-3 equivalent) age assignment for the other terrestrial mammals of the Belgrade Formation.

## Introduction

Tertiary terrestrial mammals are known from a few localities on Atlantic Coastal Plain (Tedford et al., 2004). Only four of these contain terrestrial carnivorans. The Farmingdale Local Fauna of New Jersey, initially assigned to the early Hemingfordian (Tedford & Hunter, 1984), is late late Arikareean (Ar4, MN 2–3 equivalent) in age (Emry & Eshelman, 1998). An indeterminate canid is recorded from there (Gallagher et al., 1995). The early Hemingfordian (He1, MN 4 equivalent) Pollack Farm Local Fauna of Delaware is from the Calvert Formation. It contains at least 26 species of land mammals (Emry & Eshelman, 1998). Carnivorans present are two procyonids, two borophagine canids, an ursid, and two amphicyonids. The Barstovian Chesapeake Bay Fauna of Maryland and Virginia is from the Calvert and overlying Choptank Formations (Wright & Eshelman, 1987). *Amphicyon* and the borophagine canid ?*Cynarctus marylandica* are recorded in the fauna (Tedford et al., 2004). The Lee Creek Mine Local Fauna is from the Lower to Upper Pliocene Yorktown Formation in North Carolina (Snyder, Mauger & Akers, 1983). Eshelman & Whitmore Jr (2008) documented 16 taxa from there, including two borophagine canids, an ursid, and a felid.

The Belgrade Quarry in Onslow and Jones Counties, North Carolina exposes strata of Oligocene through Pleistocene age (Locality 20 of Ward, Lawrence & Blackwelder, 1978: fig. 2; Locality 1 of Zullo, 1984). At the quarry, marine invertebrates and vertebrates have been recovered from the River Bend Formation and the overlying Belgrade Formation (e.g., Rossbach & Carter, 1991; Boessenecker, Ahmed & Geisler, 2017). The River Bend Formation is a shell hash limestone. The Belgrade Formation, which is divided into the lower Pollocksville and upper Haywood Landing Members, consists of marine shell beds, sands, and clay (Ward, Lawrence & Blackwelder, 1978).

The carnivoran jaw (NCSM 33670) described below is likely from the Belgrade Formation. At the quarry, the middle to late Oligocene River Bend Formation is mined for aggregate (McLeod & Barnes, 2008). Belgrade Formation sediments are bulldozed off of it and placed into small piles that are exposed to the elements. A mammal jaw with a p4 (NCSM 33670) was recovered while surface collecting for shark teeth and other fossils after a heavy rainstorm. Ray dental plates and shark teeth from these spoil piles include *Aetobatis* sp., *Dasyatis* sp., *Myliobatis* sp., *Rhinoptera* sp., *Galeocerdo* sp., *Hemipristis serra*, *Carcharhinus* sp., *Carcharodon angustidens*, *Carcharias* sp., *Nebrius* sp., *Isurus* sp., and *Physogaleus* sp.

The age of the Belgrade Formation is disputed. It has been assigned a late Oligocene age (Rossbach & Carter, 1991; Harris & Laws, 1997; Ward, 2002), a late Oligocene/early Miocene age (Ward, 1998), and an early Miocene age (Baum, Harris & Zullo, 1978; Zullo, Willoughby & Nystrom Jr, 1982). The Belgrade Formation in Onslow County, including the designated type section (Baum, Harris & Zullo, 1978), has been assigned to the early Miocene (Ward, Lawrence & Blackwelder, 1978; Zullo, 1984).

Terrestrial vertebrates recovered from mine spoil piles are likely earliest Miocene (late late Arikareean, Ar4, 18-23 Ma, MN 2-3 equivalent) in age (MacFadden et al., 2017). The key specimen for this determination is the entelodont *Daeodon*. The holotype of *Daeodon leidyanus* is from the late late Arikareean Farmingdale Local Fauna. However, the range of *Daeodon* in North America is early Arikareean to early Hemingfordian (Lucas, Emry & Foss, 1998). Pliocene mammal fossils are also present at Belgrade Quarry (B MacFadden, pers. comm., 2019). Therefore, it is necessary to compare NCSM 33670 with late Oligocene to Pleistocene taxa.

## Materials & Methods

Microtomography scans of the mandible were collected on a Zeiss Xradia 510 Versa nanoCT system, housed at the Analytical Instrumentation Facility at North Carolina State University. Measurements of fossil mammals at the American Museum of Natural History were made with digital calipers to the nearest 0.1 mm. Subdivision of the Arikareean North American Land Mammal Age (Ar1-4) follows Albright et al. (2008).

## Results

### Systematic paleontology

**Table:** 

Order CARNIVORA Bowdich, 1821
Family AILURIDAE Gray, 1843

### Remarks

Baskin (1998b) had previously grouped the musteloids with an elongate m2 into a single family, the Procyonidae, with two subfamilies (Procyoninae and Ailurinae). His Ailurinae contained the tribes Simocyonini and Ailurini. In the present paper, we follow the classification of Morlo & Peigné (2010) for the Ailuridae. Ailuridae is the sister taxon to the Procyonidae and Mustelidae. *Ailurus fulgens*, the red panda, is the only extant ailurid. The late Oligocene to early Miocene *Amphictis* is the sole genus of the paraphyletic Amphictinae (Morlo & Peigné, 2010) and is the stem sister taxon to the other two ailurid subfamilies, the Ailurinae and Simocyoninae.

Genus *AMPHICTIS* Pomel, 1853

**Genotypic species**: *Amphictis antiqua* (De Blainville, 1842)

### Remark

Morlo & Peigné (2010) diagnosed the genus and characterized the eight species of *Amphictis* they considered valid: *A. antiqua* (De Blainville, 1842); *A. ambigua* (Gervais, 1872); *A. milloquensis* (Helbing, 1928); *A. borbonica* (Viret, 1929); *A. schlosseri* (Heizmann & Morlo, 1994); *A. wintershofensis* Roth in (Heizmann & Morlo, 1994); *A. prolongata* (Morlo, 1996); and *A. cuspida* (Nagel, 2003). Baskin (2017) added a ninth species, *A. timicua*.

?*AMPHICTIS* sp.

**Referred specimen**. NCSM 33670, right mandible with p4, and alveoli for p1-p3, m1-m2; from Belgrade Quarry in Jones County, North Carolina, USA.

**Figure 1:**
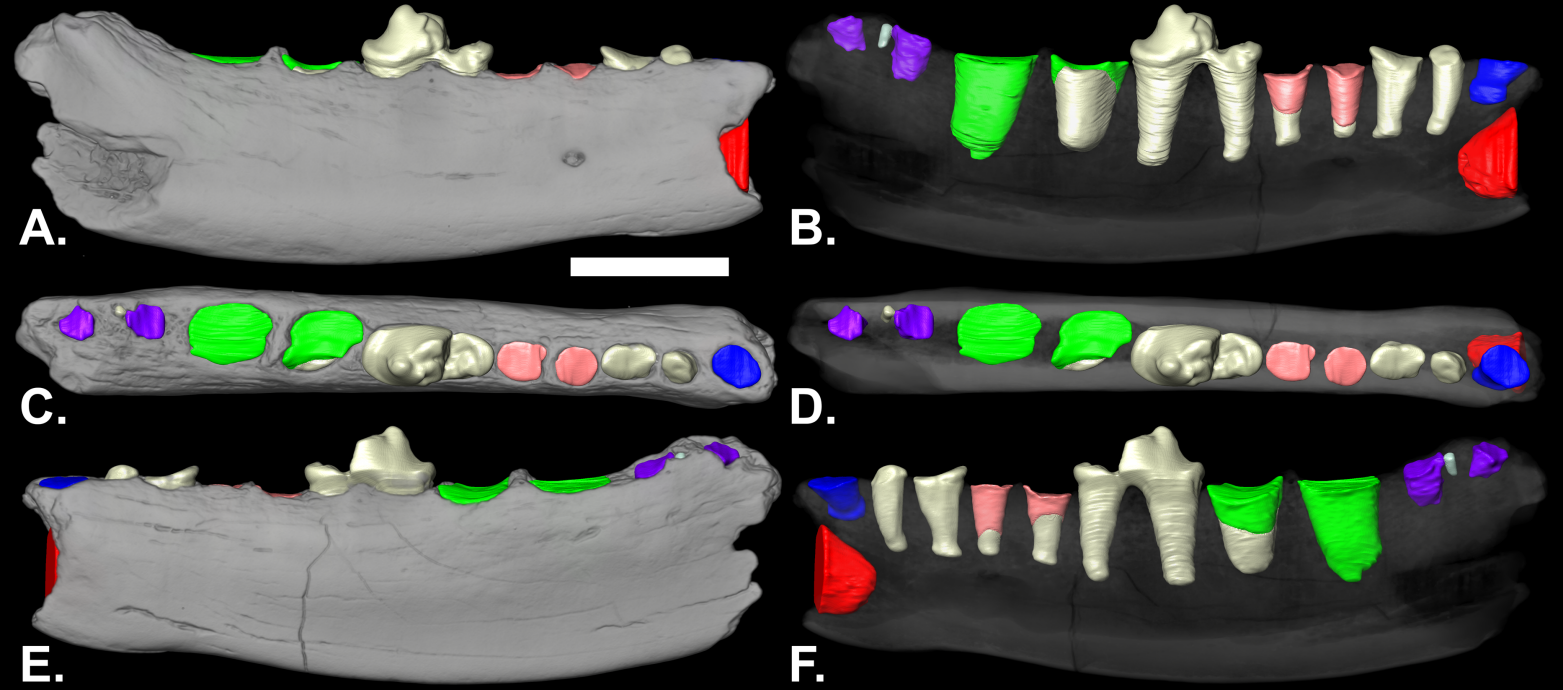
Lateral (A & B), occlusal (C & D) and medial (E & F) views of volumetric reconstruction of NCSM 33670. Mandibular bone (in gray) has been rendered semitransparent in B, D, and F to display root morphology. Preserved dental elements (root and crown fragments) are tan. Alveolar casts are rendered in red (canine), blue (p1), pink (p3), green (m1) and purple (m2). A–D, anterior is to the right; E & F, anterior is to the left. Scale bar = 1 cm.

**Description:** The mandible extends from posterior to the canine to the posterior alveolus of m2 (Fig. 1). It is somewhat shallower beneath the p3 (Table 1). Posterior from beneath the p3, the ventral border is gently convex posterior to beneath the p3. The mental foramen is below the anterior root of the p3, at approximately half the depth of the ramus. The coronoid process is missing. Length-wise cracks on the surface, porous bone, and polish on parts of the surface are indications of weathering and perhaps transport before burial.

**Table 1:** Measurements in mm on NCSM 33670.

p2 L	5.7
p3 L	6.1
p4l	8.1
p4 W	4.0
m1 L	10.9
m2 L	6.9
p2-m1 L	31.6
p2-m2 L	39.5
p1-m2 L	42.8
Depth p2	10.75
Depth m1	12.5

**Notes.**

L length W width Depth p2 and Depth 1m depth of mandible below p2 and m1, respectively

The p1 is represented by a single, shallow alveolus. The roots are preserved in the p2 alveoli. The p3 alveoli contain the distal tips of the roots. The p4 is the only tooth in the mandible with crown elements preserved (Fig. 1). The relatively intact roots of p2 and p4 are preserved, as are the apical-most portions of both of the roots of p3 and much of the anterior root of m1 (Figs. 1B and 1F). The main cusp of the p4 is broken anterior to the midline of the tooth. There is a distinct posterior accessory cusp on the external margin of the main cusp. The anterior cingulid is better developed antero-internally than antero-externally. The posterior cingulid is well developed.

**Figure 2:**
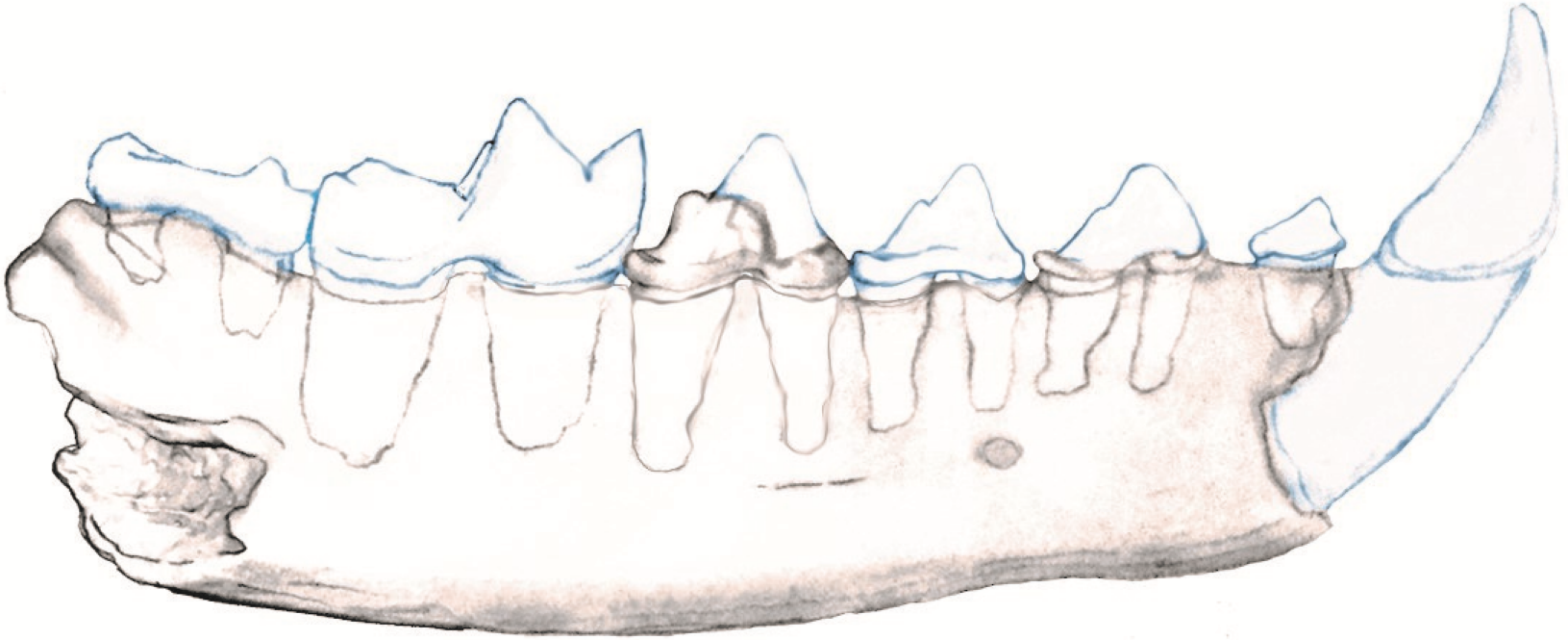
Reconstruction of NCSM 33670 showing mandible with c1-m2.

The posterior root of the m1 is larger than the anterior root. The m2 is double rooted and situated on the slope of the ascending coronoid process. In the nano-CT volumetric reconstruction (Figs. 1B and 1F), the posterior root appears smaller than the anterior root. However, the bone surface surrounding that alveolus is eroded. The reconstruction in Fig. 2 is based on illustrations of *Amphictictis schlosseri* (Heizmann & Morlo, 1994) and *A. milloquensis* (Cirot & Wolsan, 1995), and casts of *A. winterhofensis* in the AMNH. As reconstructed, the two roots were nearly identical in depth, with the posterior alveolus extended upward and sloped toward the anterior margin of where the coronoid process would have been. Therefore, the m2 posterior root was a much more substantial structure that extended both distally and occlusally than what is preserved. There is a third small accessory rootlet between the anterior and posterior root.

## Discussion

The presence of only a single, relatively non-diagnostic tooth, the p4, and the possibility that the jaw may not be late late Arikareean make a confident generic determination difficult. If an m3 were present, the root or its alveolus would have been evident in the microtomography scan (Fig. 1). The absence of an m3 indicates NCSM 33670 is a member of the Musteloidea—the Mephitidae, Ailuridae, Mustelidae, and Procyonidae (Wolsan, 1993; Wang, McKenna & Dashzeveg, 2005). The highly sloping distal molar, transitioning to the coronoid process out of the strict occlusal plane, is another feature seen only in musteloids, and immediately identifies the jaw as neither a canid nor an amphicyonid. An elongate m2 and an elongate m1 talonid (as suggested by the larger posterior m1 alveolus) are characteristic of the Ailuridae and Procyonidae. NCSM 33670 was therefore compared with late Oligocene and early Miocene basal musteloids, mustelids, ailurids, and procyonids, as well as with middle Miocene and younger ailurids and other musteloids.

NCSM 33670 compares best with *Amphictis*, the earliest and most primitive member of the Ailuridae (Morlo & Peigné, 2010). *Amphictis* is known from the late Oligocene (Arvernian, MP 28) to early Miocene (Orleanian, MN 3-4) of Europe (Heizmann & Morlo, 1994; Cirot & Wolsan, 1995; Morlo, 1996), the middle Miocene (late Orleanian, MN 5 or Astaracian, MN 6) of Turkey (Nagel, 2003), and the late early Miocene (early Hemingfordian, MN 4 equivalent) of North America (Tedford et al., 1987; Baskin, 2017). A possible earlier North American occurrence is AMNH 81029 (misnumbered AMNH 81040 in Baskin, 2017), a mandible from the late late Arikareean (AR4, MN 2-3 equivalent) of Nebraska identified as *Bassariscus* sp. (Cook & Macdonald, 1962). Baskin (2004) considered AMNH 81029 *Amphictis*-like.

**Figure 3:**
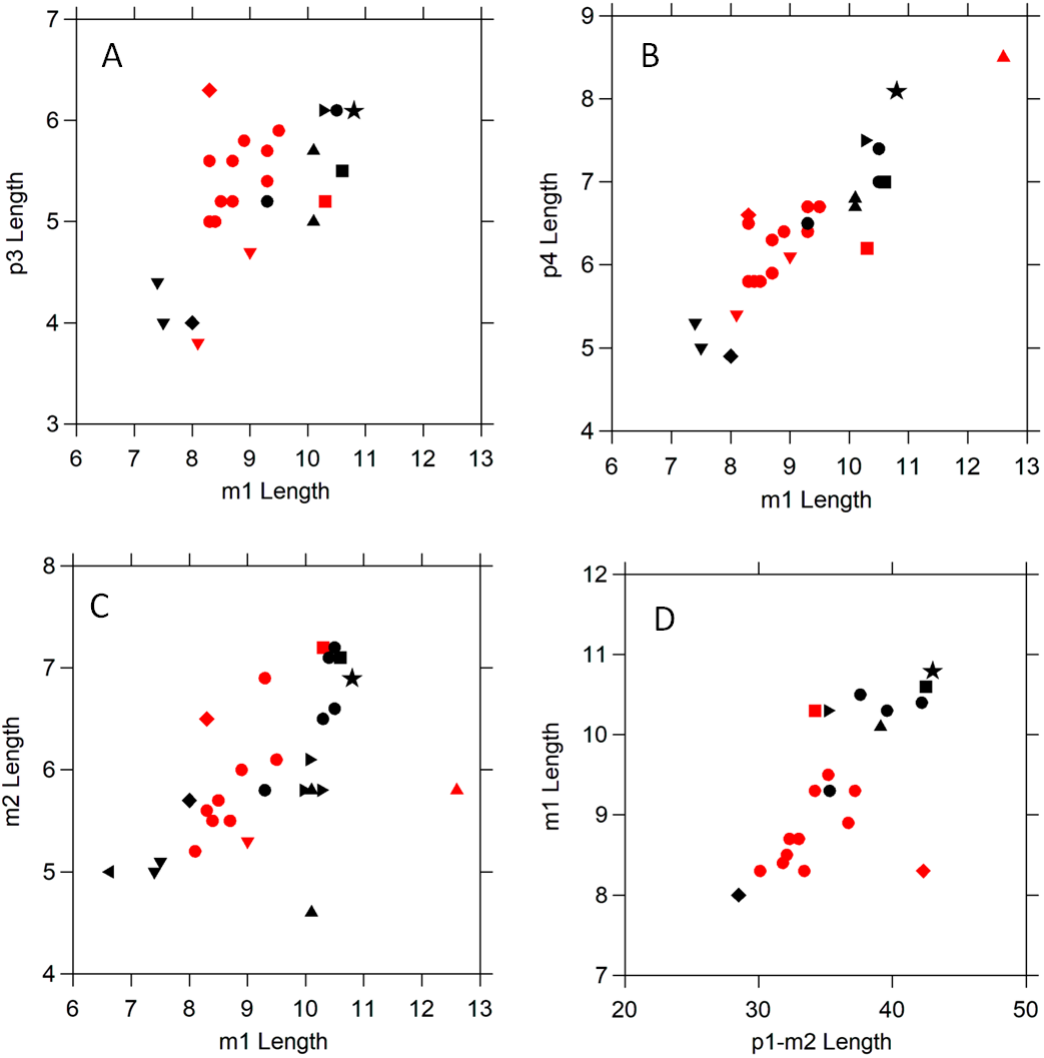
Scatter plots comparing tooth dimensions of selected early and middle Miocene ailurids and procyonids. Symbols are as follows: black star ⋆, NCSM 33670; black •, *Amphictis ambigua*^1^; black ►, *Amphictis schlosseri*^1^; black ▴, *Amphictis milloquensis*^2^; red •, *Amphictis winterhofensis*^3^; red ▴, *Amphictis cuspida*^4^; black ▾, *Amphictis timicua*^5^; red ▾, *Amphictis* cf. *timicua*^5^; red ■, *Actiocyon parverratis*^5^; black ■, *Alopecocyon goeriachensis*^6^; black ⧫, *Bassaricyonoides phyllismillerae*^7^; red ⧫, ?*Edaphocyon palmeri*^7^. Sources of data: ^1^Heizmann & Morlo (1994); ^2^Cirot & Wolsan (1995); ^3^Dehm (1950); ^4^Nagel (2003); ^5^Baskin (2017); ^8^Smith, Czaplewski & Cifelli (2014); ^6^Peigné (2012); ^7^Baskin (2003).

The m1 length of NCSM 33670 is greater than that of *Amphictis antiqua*, *A. borbonica*. *A. wintershofensis*, and *A. timicua*; similar to that of *A. ambigua*, *A. milloquensis*, and *A. schlosseri*; and less than that of *A. cuspida* (Heizmann & Morlo, 1994; Cirot & Wolsan, 1995; Nagel, 2003; Baskin, 2017). The teeth have similar length proportions such as the ratios of p3, p4, and m2 to m1 (Table 2, Fig. 3). The middle Miocene (MN 6) *A. cuspida* from Çandir, Turkey is the latest and largest species (Nagel, 2003). It is known from a lower jaw with p4-m2. *Amphictis cuspida* differs from other *Amphictis*, including NCSM 33670, in having a shorter m2 as compared to the m1 (Table 2, Fig. 3). The only other specimen in Fig. 3 with such a relatively short m2 is the holotype of *Plesictis milloquensis* from the Late Oligocene (MP 29) of La Milloque in southwestern France (Helbing, 1928) which Cirot & Wolsan (1995) transferred to *Amphictis*. The additional specimen they identified as *A. milloquensis* (Cirot & Wolsan, 1995: Fig. 1) has the m2:m1 (Table 2) ratio the same as that of the early Miocene (MN 1–MN 2a) *A. schlosseri* from the Molasse basin and Mainz basin of southwestern Germany (Heizmann & Morlo, 1994). *Amphictis* from the Hemingfordian of Florida and Nebraska (Baskin, 2017) is much smaller than NCSM 33670. The *Bassariscus*-like posterior ramus with alveoli for the posterior root of m1 and for two roots of an elongate m2 from the Hemingfordian Pollack Farm of Delaware is similar to these Florida and Nebraska *Amphictis* (Emry & Eshelman, 1998; Baskin, 2003; Baskin, 2017). NCSM 33670 is near the high end of the alveolar measurements of the species of *Amphictis* plotted in Fig. 3. Measurements of *Amphictis ambigua* from Quercy at Pech du Fraysse (MP 28) are most similar to those of NCSM 33670. The p4 of NCSM 33670 is similar to that of *A. schlosseri* (Heizmann & Morlo, 1994: plate 2) in having well-developed anterior and posterior cingulids and an externally situated posterior accessory cusp. The posterior cingulid is wider in NCSM 33670.

**Table 2:** Measurements in mm of of taxa discussed in the text.

	n	p4 L	m1 L	m2 L	m2 L/m1 L
NCSM 33670	1, 1, 1	8.1	10.8	6.9	0.68
*Amphictis antiqua*^1^	?, 1, 1		9.3	5.9	0.63
*Amphictis.ambigua*^1^	3, 5, 5	7.0 ± 0.45	10.2 ± 0.51	6.6 ± 0.56	0.65 ± 0.031
*Amphictis.milloquensis*^2^	1, 1, 1	5.7	10.1	4.6	0.45
*Amphictis milloquensis*^3^	1, 1, 1	5.0	10.1	5.8	0.57
*Amphictis borbonica*^5^	1, 1, 1	6.2	9.0	5.2	0.58
*Amphictis borbonica*^1^	?, 1, 1		9.4	5.2	0.55
*Amphictis schlosseri*^1^	1, 3, 3	7.5	10.1 ± 0.15	5.9 ± 0.17	0.58
*Amphictis wintershofensis*^4^	10,11, 10	6.3 ± 0.37	8.7 ± 0.47	5.8 ± 0.47	0.66 ± 0.035
*Amphictis prolongata*^5^	1, 1, 1	6.5	8.8	6.0	0.68
*Amphictis cuspida*^6^	1, 1, 1	8.5	12.6	5.8	0.46
*Amphictis timicua*^7^	2, 2, 2	5.0, 5.3	7.5, 74	5.1, 50	0.68
*Actiocyon parverratis*^8^	1, 1, 1	6.2	10.3	7.2	0.69
*Alopecocyon goeriachensis*^9^	1, 1, 1	7.0	10.6	7.1	0.66
*Actiocyon* sp.^10^	1, 1, 1	7.0	15.4	11.8	0.76
*Franconicits humilidens*^4^	11, 16, 12	6.5 ± 0.32	8.9 ± 0.43	4.7 ± 0.31	0.54 ± 0.026
*Peignictis pseudamphictis*^11^	?, 1, 1		5.3	2.5	0.47
*Floridictis kerneri*^7^	10, 10, 3	7.5 ± 0.53	11.8 ± 0.37	2.8 ± 0.23	0.24

**Notes.**

n number of specimens of p4, m1, m2

Sources of data:

1 Heizmann & Morlo (1994)

2 NMB LM 554, Cirot & Wolsan (1995)

3 FSP LM 1969 MC 2, Cirot & Wolsan (1995)

4 Dehm (1950)

5 Morlo (1996)

6 Nagel (2003)

7 Baskin (2017)

8 Smith, Czaplewski & Cifelli (2014)

9 Peigné (2012)

10 F:AM 25212, Ash Hollow Formation, Nebraska

11 De Bonis, Gardin & Blondel (2019)

**Figure 4:**
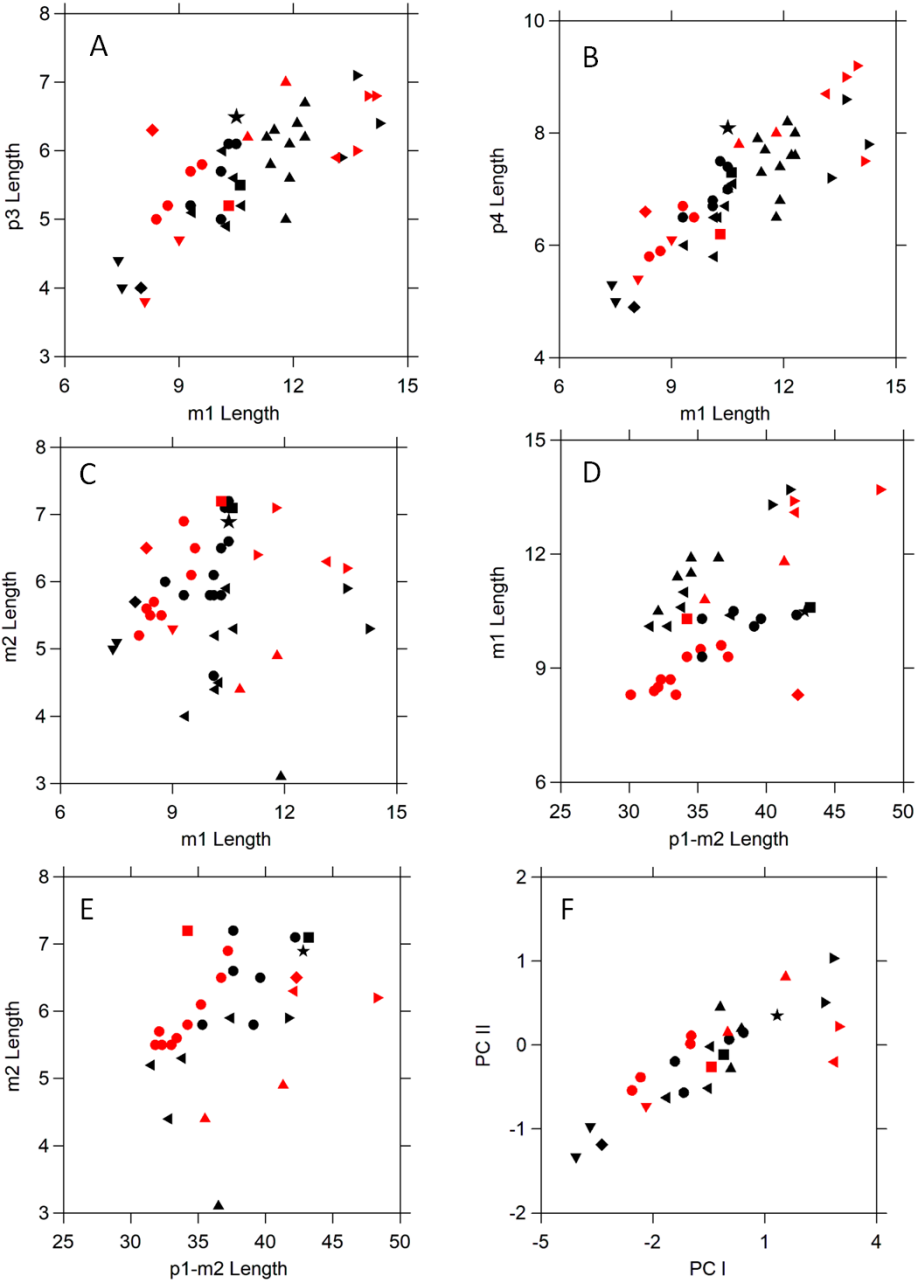
Scatter plots comparing tooth dimensions of selected *Amphictis* and early and middle Miocene mustelids, and procyonids. Symbols are as follows: black star ⋆, NCSM 33670; black •, *Amphictis* spp.^1^; red •, *Amphictis winterhofensis*^2^; black ▾, *Amphictis timicua*^3^; red ▾, *Amphictis* cf. *timicua*^3^; red ■, *Actiocyon parverratis*^4^; black ■, *Alopecocyon goeriachensis*^5^; black ⧫, *Bassaricyonoides phyllismillereae*^6^; red ⧫, ?*Edaphocyon palmeri*^6^; black ▴, *Floridictis kerneri*^3^; red ►, *Zodiolestes*^3^; black ◂, *Oligobunis*^3^; red ►, *Brachypsalis*^3^; black ◂, *Promartes* spp. 3; red ◂, *Parabrachypsalis janisae*^3^. Sources of data: ^1^Heizmann & Morlo (1994), Cirot & Wolsan (1995); ^2^Dehm (1950); ^3^Baskin (2017); ^4^Smith, Czaplewski & Cifelli (2014); ^5^Peigné (2012); ^6^Baskin (2003).

*Alopecocyon* from the middle Miocene of Europe (MN 6–MN 7/8) is closely related to *Amphictis* (De Beaumont, 1976; Morlo & Peigné, 2010). De Beaumont (1976) noted that the lower dentition of *Alopecocyon goeriachensis*, the type species of the genus, was difficult to distinguish from that of *Amphictis*. Wolsan (1993) synonymized *Alopecocyon* with *Amphictis*. Plots of dental measurements (Figs. 3 and 4) support Wolsan. However, Morlo & Peigné (2010) noted that this had not been accepted by subsequent authors. Webb (1969), following a suggestion from D. E. Savage, synonymized *Actiocyon* (Stock, 1947) from the Clarendonian Nettle Spring Fauna of the Caliente Formation of California with *Alopecocyon*. Smith, Czaplewski & Cifelli (2014) distinguished North American *Actiocyon* from Eurasian *Alopecocyon* by morphology of the cusps on m2. *Actiocyon parverratis* from the early Barstovian (MN 6 equivalent) of Nevada (Smith, Czaplewski & Cifelli, 2014) and *Actiocyon* sp. **from the late Clarendonian Ash Hollow Formation of Nebraska have an elongate m2 relative to m1, but only the Ash Hollow specimen has the m2 significantly more elongate than** **Amphictis** (Table 2). More derived ailurids such as *Pristinailurus* from the late Miocene to early Pliocene Gray Fossil Site in Tennessee (Wallace & Wang, 2004; Wallace, 2011) have a more complex p4 and an even more elongate m2. Tooth measurements (Table 2, Figs. 3 and 4) indicate NCSM 33670 is more similar to the larger species of *Amphictis* than to *Alopecocyon* or other ailurids.

In North America, procyonids are known from the Hemingfordian of Florida, Nebraska, and Delaware. The procyonid from the early Hemingfordian Pollack Farm of Delaware (Emry & Eshelman, 1998) is an M1 compared to *Edaphocyon* (Wilson, 1960). The only mandible attributed to this genus, the Hemingfordian ?*E. palmeri* (Baskin, 2003), has an elongate m2 (Table 2), but differs from *Amphictis* and NCSSM 33670 in other dental proportions (Figs. 3 and 4). The Hemingfordian *Bassaricyonoides phyllismillerae* (Baskin, 2003) has p4 much wider especially posteriorly and with a more prominent posterior accessory cusp on the postero-external margin. The Barstovian to Recent *Bassariscus* and the Barstovian *Probassariscus* are smaller than NCSM 33670 and have a more elongate m2. In the eastern U.S. procyonids occur in Clarendonian and younger sites of Florida. The only extant procyonid that may have been present in North Carolina in the Pliocene and Pleistocene is the raccoon *Procyon*. In *Procyon* the m1 and m2 are more or less equal in length (Wright & Lundelius Jr, 1963) and the p4 is noticeably wider posteriorly and has a more laterally situated posterior accessory cusp.

*Plesictis* is a small mustelid from the late Oligocene and early Miocene of Europe (De Bonis, Gardin & Blondel, 2019). *Mustelavus priscus* (Clark, 1936) is a musteloid from the late Eocene of western North America (Baskin, 1998a). Simpson (1946, p.13) considered *Mustelavus* a “probable synonym” of *Plesictis*. Because of similarities with the type of *M. priscus*, Macdonald (1970), following Simpson, identified an anterior mandible from the early Arikareean (Ar2) of South Dakota as *Plesictis* sp. Tedford et al. (2004) noted this occurrence as an immigration event for *Plesictis*. However, the generic identity of this South Dakota specimen has not been demonstrated. Wang, McKenna & Dashzeveg (2005) include the Chadronian to Orellan *Mustelavus priscus*, *Plesictis*, and several other taxa as basal musteloids. The Orleanian *Plesictis*? *humilidens* (Dehm, 1950) is from Wintershoff-West. Wolsan (1993) made it the type species of *Franconictis*, which he classified as a mustelid that was the sister taxon of *Stromeriella*. Both have a relatively long m2. *Franconicits humilidens* has similar dimensions (Dehm, 1950: Table 11) to those plotted in Figs. 3 and 4 for *Amphictis winterhofensis*, other than a somewhat shorter m2 length relative to m1 (Table 2).

The early Oligocene *Peignictis pseudamphictis* is an enigmatic musteloid known from a posterior ramus with m1 (De Bonis, Gardin & Blondel, 2019). The posterior alveolus of the m2 is smaller than the anterior one. It is much smaller than *Amphictis* (Table 2) and has an m1 morphology more like that of *Mustelictis* (Bonis de, Gardin & Blondel, 2019). Although the m2 is relatively long, the m2:m1 ratio is less than that of *Amphictis* or NCSM 33670.

The Oligobuninae are the sister group of the neomustelids (Baskin, 1998a; Baskin, 2017; Valenciano et al., 2016). They, neomustelids, and procyonids are derived relative to ailurids in lacking an alisphenoid canal (Wang, McKenna & Dashzeveg, 2005). *Promartes*, the earliest oligobunine, is known from the late early Arikareean (Ar3) to Hemingfordian. Its species are similar in size to NCSM 33670 (Fig. 4). They and the other oligobunines such as *Floridictis*, *Zodiolestes*, *Oligobunis*, *Brachypsalis*, and *Parabrachypsalis* (Baskin, 2017) differ from NCSM 33670 in having a relatively shorter m2 and a relatively longer m1 (Table 2, Fig. 4).

## Conclusions

The late Oligocene and early Miocene are marked by the immigration of carnivorans from Eurasia to North America (Tedford et al., 2004: fig. 6.3). Among the musteloids, *Mustelictis* from the early Oligocene (MP 22, 32 Ma) of Quercy, France (De Bonis, 1997) is one of the earliest stem mustelids (Wang, McKenna & Dashzeveg, 2005). *Corumictis wolsani*, from the early Arikareean (Ar1, MP 24 equivalent, 30–28 Ma and Ar2) of Oregon, is the earliest stem mustelid in North America and is an immigrant taxon (Paterson et al., 2020). *Promartes* is the only oligobunine mustelid known from the Arikareean. Whether the Oligobuninae are autochthonous or allochthonous is unresolved. Crown clade mustelids and procyonids first appear in North America during the Hemingfordian. The procyonids are derived from a European early Miocene taxon such as *Broiliana* (Baskin, 1998b). Previously the earliest ailurids in North America were from the Hemingfordian of Florida and Nebraska (Baskin, 2017). NCSM 33670 is most similar to the late Oligocene *Amphictis ambigua* (MP 28) or the early Miocene *A. schlosseri* (MN 1–MN 2a). Based on marine invertebrates, as noted above, the Belgrade Formation has been considered late Oligocene (Chattian or Chickasawhayan) to early Miocene (Aquitanian) in age. MacFadden et al. (2017) propose a late late Arikareean (AR 4, MN 2–3 equivalent) age assignment for the mammals from the Belgrade Formation, In any case, an Arikareean (late Oligocene to early early Miocene) age assignment supports NCSM 33670 being an immigrant from Eurasia and the earliest record of the Ailuridae in North America.

##  Supplemental Information

10.7717/peerj.9284/supp-1 Supplemental Information 1 Ailuridae dental measurements used to construct Figure 3

10.7717/peerj.9284/supp-2 Supplemental Information 2 Mustelida dental measurements used to construct Figure 4

## Institutional abbreviations

AMNH: American Museum of Natural History, New York, New York
F:AM: Frick Collection of fossil mammals in the AMNH
NCSM: North Carolina Museum of Natural Sciences

## Anatomical Abbreviations

p: lower premolar
m: lower molar
L: length
W: width

## Additional Information and Declarations

### Field Study Permissions

The following information was supplied relating to field study approvals (i.e., approving body and any reference numbers): Permission to collect was granted by the operators of the Martin Marietta quarry at Belgrade, North Carolina.

### Data Availability

The following information was supplied regarding data availability: The raw measurements used to construct Figures 3 and 4 are available in the Supplemental Files. NCSM 33670 is accessioned in the North Carolina Museum of Natural Sciences, Raleigh, North Carolina. AMNH specimens are available in the collection of the Department of Paleontology, American Museum of Natural History, New York, New York (accession numbers: AMNH 81029, AMNH 81040). The 3D model is available at Morphosource: https://www.morphosource.org/Detail/ProjectDetail/Show/project_id/973.
